# Personal health promotion at US medical schools: a quantitative study and qualitative description of deans' and students' perceptions

**DOI:** 10.1186/1472-6920-4-29

**Published:** 2004-12-06

**Authors:** Erica Frank, Joan Hedgecock, Lisa K Elon

**Affiliations:** 1 Dept. of Family and Preventive Medicine Emory University School of Medicine 49 Jesse Hill Jr. Drive, Atlanta, GA 30303, USA; 2 American Medical Student Association/Foundation 1902 Association Drive, Reston, VA 20191, USA; 3 Department of Biostatistics Emory University Rollins School of Public Health 336 G.C. Rollins School of Public Health, Atlanta, GA 30322, USA

## Abstract

**Background:**

Prior literature has shown that physicians with healthy personal habits are more likely to encourage patients to adopt similar habits. However, despite the possibility that promoting medical student health might therefore efficiently improve patient outcomes, no one has studied whether such promotion happens in medical school. We therefore wished to describe both typical and outstanding personal health promotion environments experienced by students in U.S. medical schools.

**Methods:**

We collected information through four different modalities: a literature review, written surveys of medical school deans and students, student and dean focus groups, and site visits at and interviews with medical schools with reportedly outstanding student health promotion programs.

**Results:**

We found strong correlations between deans' and students' perceptions of their schools' health promotion environments, including consistent support of the idea of schools' encouraging healthy student behaviors, with less consistent follow-through by schools on this concept. Though students seemed to have thought little about the relationships between their own personal and clinical health promotion practices, deans felt strongly that faculty members should model healthy behaviors.

**Conclusions:**

Deans' support of the relationship between physicians' personal and clinical health practices, and concern about their institutions' acting on this relationship augurs well for the role of student health promotion in the future of medical education. Deans seem to understand their students' health environment, and believe it could and should be improved; if this is acted on, it could create important positive changes in medical education and in disease prevention.

## Background

Our purpose was to describe both typical and outstanding personal health promotion environments experienced by medical students in U.S. medical schools. Our interest in health promotion among medical students was based on compelling data showing that physicians who have healthy personal habits are more likely to encourage patients to adopt related habits [1]. However, despite the clear possibility that promoting medical student health should therefore be an innovative, efficient, and effective way to improve patient outcomes, no one has examined the extent to which Deans or students believe that this concept is enacted in medical school.

## Methods

We collected information through four different modalities: a literature review, a written survey of medical school Deans and students, focus groups of preclinical and clinical medical students and dean, and site visits at and interviews with medical schools with reportedly outstanding student health promotion programs.

### Medical student and dean surveys

There were 17 respondents to the Dean Survey (DS), representing 12 of the 16 schools in the nationally representative Healthy Doc (HD) project [2]. Two deans responded from Mercer, RWJ/UMDNJ, Tulane, UCLA, and University of Pennsylvania, while Colorado, Creighton, Emory, Georgetown, Loma Linda, Medical College of Georgia, and University of Rochester each had one dean respond. It was not always clear whether the Dean of Curriculum or the Dean of Student Affairs was the respondent, therefore we did not differentiate in the analyses by dean type. We also compared Deans' responses with responses (83% response rate) from the 1336 medical students in the Class of 2003 in these Deans' schools, as they were about to begin on wards. All medical students in that class were eligible to complete a self-administered questionnaire covering personal and professional health promotion topics. Our sample of schools was designed to be representative of all U.S. medical schools in our geographic distribution, age (our freshman average was 24 vs. 24 nationally), school size (our schools averaged 563 medical students/school vs. 527 nationally), NIH research ranking (our average was 64 vs. 62 nationally), private/public school balance (51% in private schools vs. 41% nationally), under-represented minorities (13% Blacks, Hispanics, and Native Americans, vs. 11% nationally), and gender (45% women vs. 43% nationally)^5–7 ^Methodology for gathering medical student data in HD has been more fully described elsewhere [2]. DS data were collected between February 2002 and April 2003.

In analyses comparing DS and HD data, DS schools with two respondents were first averaged so that each school is represented by one value (since repeated measures analysis was not available for the desired analyses). Variation between deans representing a school was quite low for all but one pair. By averaging for the five dean pairs (and consequently having a sample size of 12 rather than 17), the tests are conservative. Student opinion scores were also averaged for each of the twelve schools from which we received Dean responses; these averages were then correlated with the Dean's scores using Spearman's correlation method. For questions with fairly uniform responses by either Deans or students, Wilcoxon's Signed Rank Test was used to test if there were consistent differences between student and Dean opinion. The two variables to be correlated were ordinal variables, each with 5 levels. The type of correlation method was therefore limited to a non-parametric method. Additionally, the raw student data was clustered by school, requiring methods suitable for correlated data. Since the non-parametric method needed is not available for correlated data, we determined that the best method was to take the student mean values at each school to correlate with the dean values. While this ignored the student variability within school, this deficit was balanced by the fact that the much smaller n would require much stronger evidence of a relationship to evince a significant result.

Deans were also asked to rate their school relative to other schools. To compare these ratings to students' opinions, schools were ranked using their mean student scores on each question related to prevention and healthy activities encouraged by the school. All 16 schools in the HD cohort were used in the ranking process (1 = highest, 16 = lowest), not just the 12 schools represented by the responding deans, as the 16 were the intended sample, and are representative of US medical schools [2]. Therefore, the twelve schools for which we have Dean data could have rank values between 1 and 16. For Deans' survey questions without comparative HD data, only simple descriptive statistics are presented.

### Medical student and dean focus groups

For our focus groups (conducted in 2002), we identified opportunities where there would be a wide and nationally representative range of medical schools. The first focus group was convened at the AMSA Chapter Officers' Training Conference (COC) attended by student leaders (primarily rising second years) from every U.S. osteopathic and allopathic medical school. AMSA invited a random sampling of those attending the COC to participate in the focus group. Since the first focus group of students attracted 10 first and second year students, the second focus group was a random sample of 12 clinical students; both student focus groups had an even gender mix. Because Philadelphia has so many medical schools (five), we sampled for the second focus group from those Philadelphia students who were listed in AMSA's membership database. Deans of Primary Care were invited to the third focus group convened at the annual conference of the Association of American Medical Colleges. AMSA used the list of Primary Care Deans and invited a random sample of them to attend the focus group; four attended. An outside contractor (Bennett, Petts & Blumenthal) assisted AMSA in developing the focus group guide, conducted all three focus groups, transcribed the conversations and analyzed the notes for trends in responses.

### Site visits and interviews

In 2002–2003, we identified medical school campuses with intensive programs in medical student well-being through literature and web searches, recommendations from project advisory panel members, results from the Association of Academic Health Centers' American Network of Health Promoting Universities assessment, and participants in the HRSA-funded UME-21 project. Site visits and in-depth interviews were conducted using a protocol which sought information and recommendations on the following topics:

• Student well-being programming, including the policies, activities, and evaluation for such efforts as stress reduction, exercise, diet, and mentoring.

• Prevention in the curriculum using the *Healthy People 2010 *objectives and how the various topics are integrated, taught, and evaluated.

• Deans' office support (including financial) for prevention in the curriculum and student wellness activities.

• Student assessments and recommendations regarding their schools' efforts.

## Results

### Survey of Deans and medical students

Most surveyed Deans reported that their schools generally support students' health, though fewer Deans believe that their school encourages healthy eating (Table 1). Both Deans and students rate their programs rather positively, and their responses are very highly correlated, though Deans consistently rate their programs even more positively than do students (Table 2).

**Table 1:** Deans' beliefs regarding their schools' student health promotion efforts

	**% (n)**				
	**Strongly agree**	**Agree**	**Neither agree nor disagree**	**Disagree**	**Strongly disagree**

1.1 Overall, our medical school encourages students to lead healthy lives.	35 (6)	53 (9)	6 (1)	6 (1)	0
1.2 Our medical school curriculum emphasizes preventive medicine in medical practice.	29 (5)	53 (9)	12 (2)	6 (1)	0
1.3 Our medical school encourages extracurricular activities that promote medical students' health.	35 (6)	35 (6)	18 (3)	6 (1)	6 (1)
1.4 Our medical school tries to minimize student stress.	41 (7)	41 (7)	12 (2)	0	6 (1)
1.5 Our medical school has a good system to help students cope with stress.	29 (5)	47 (8)	18 (3)	6 (1)	0
1.6 Our medical school encourages students' healthy eating.	12 (2)	47 (8)	35 (6)	6(1)	0
1.7 Our medical school encourages students to exercise.	24 (4)	53 (9)	12 (2)	6 (1)	6 (1)
1.8 Our medical school discourages students from smoking	41 (7)	47 (8)	12 (2)	0	0

**Table 2:** Mean scores* for and correlation coefficients ^¶ ^between Deans' and students' responses to statements concerning health promotion at medical school.

	**Deans' mean score**	**Students' mean score**	**r**	**p-value**
Overall, our medical school encourages students to lead healthy lives.	1.8	2.5	.87	.0002
Our medical school curriculum emphasizes preventive medicine in medical practice.	1.9	2.3	.51	.0912
Our medical school encourages extracurricular activities that promote medical students' health.	2.1	2.7	.54	.0681
Our medical school tries to minimize student stress.	1.8	3.0	.91	<.0001
Our medical school has a good system to help students cope with stress.	2.0	2.9	.70	.0110
Our medical school encourages students' healthy eating.	2.3	3.1	.74	.0064
Our medical school encourages students to exercise.	2.1	2.9	.48	.1139

*Responses were scored 1 for "strongly agree", continuing to 5 for "strongly disagree". Therefore higher scores indicate less agreement with the statement.

^¶ ^Spearman's correlation coefficients.

Deans were essentially unanimous in agreeing that faculty members should model healthy behaviors, and that schools should promote health with their students (Table 3). However, Deans felt less strongly regarding the need for more training in prevention for primary care physicians, or that a physician must have a healthy lifestyle to effectively counsel patients on healthy lifestyles (Table 3). Students also agreed with these statements, but generally to a lesser extent than Deans (Table 4).

**Table 3:** Deans' opinions on the role medical schools and physicians should play in promoting healthy behaviors/prevention

	**% (n)**				
	**Strongly agree**	**Agree**	**Neither agree nor disagree**	**Disagree**	**Strongly disagree**

1.9 Medical school faculty members should set a good example for medical students by practicing a healthy lifestyle.	59 (10)	35 (6)	6 (1)	0	0
1.10 Medical schools should encourage students and residents to practice healthy lifestyles.	65 (11)	35 (6)	0	0	0
1.11 Primary Care physicians need more training in prevention.	29 (5)	59 (10)	12 (2)	0	0
1.12 In order to effectively encourage patient adherence to a healthy lifestyle, a physician must adhere to one him/herself.	18 (3)	65 (11)	12 (2)	6 (1)	0

**Table 4:** Dean and student opinions on the need for schools and faculty to promote healthy lifestyles, the need for more prevention training, and the connection between a physician's healthy lifestyle and his/her counseling efficacy.

	**Deans' mean score**	**Students' Mean score**	**Wilcoxon signed rank test p-value**
Medical school faculty members should set a good example for medical students by practicing a healthy lifestyle.	1.4	2.1	.0015
Medical schools should encourage their students and residents to practice healthy lifestyles.	1.3	1.9	.0015
Doctors need more training in prevention.	1.8	2.1	.0342
In order to effectively encourage patient adherence to a healthy lifestyle, a physician must adhere to one him/herself.	2.1	2.2	.3804

Three-quarters of Deans believed that their medical schools' attitude toward alcohol was that drinking in moderation was acceptable, though students had more mixed impressions about schools' alcohol attitudes (Table 5). Deans believed that their schools did average or better on nearly all health promotion activities (Table 6), and students' and Deans' assessments of their schools are highly correlated (Table 7).

**Table 5:** Deans' and students' impressions of their medical schools' attitudes about alcohol use

	**Deans**	**Students**
No obvious attitude	18%	25%
Students shouldn't drink at all	6%	13%
Drinking in moderation is acceptable	76%	50%
Drinking is a good release	0%	11%

**Table 6:** Deans' comparisons of their medical school vs. other medical schools

	**"My school does this (circle choice) compared to other schools."**				
	
	Much more	Some-what more	An average amount	Somewhat less	Much less
	
	%(n)				
3.1 Encourages students to lead healthy lives.	24 (4)	29 (5)	41 (7)	6 (1)	0
3.2 Emphasizes preventive medicine in medical practice.	6 (1)	29 (5)	53 (9)	12 (2)	0
3.3 Encourages extracurricular activities that promote medical students' health.	24 (4)	18 (3)	41 (7)	18 (3)	0
3.4 Encourages students to exercise.	6 (1)	38 (6)	38 (6)	19 (3)	0
3.5 Helps students minimize/cope with stress.	24 (4)	47 (8)	18 (3)	6 (1)	6 (1)
3.6 Discourages students from smoking.	12 (2)	41 (7)	41 (7)	6 (1)	0
3.7 Discourages drinking as a release for students.	6 (1)	12 (2)	65 (1)	18 (4)	0
3.8 Encourages students' healthy eating.	6 (1)	24 (4)	65 (11)	6(1)	0

**Table 7:** Comparing Dean's perceptions of their school's health promotion in relation to that of other medical schools* with school rankings based on students' opinions.

"My school	**r**^¶^	**p-value**
...encourages students to lead healthy lives."	.78	.0026
...emphasizes preventive medicine in medical practice."	.45	.1433
...encourages extracurricular activities that promote medical students' health."	.70	.0118
...encourages students to exercise."	.77	.0051
...helps students minimize/cope with stress."	.77/.75^φ^	.0033/.0047
...encourages students' healthy eating."	.68	.0151

*In which response possibilities were: much less, less, average, more, much more.

^¶ ^Spearman's correlation coefficient.

^φ^The Deans' survey asked one question that queried both minimizing and coping with stress, while students were asked one about each aspect of stress. Correlations are presented for minimizing stress and coping with stress, respectively.

We also asked a few narrative questions of the Deans only. Deans indicated that Student Affairs and Student Health offices most often had responsibility for handling medical student wellness (responses of 10 and 4 Deans, respectively). Funds for student wellness activities primarily came from student fees and University budgets (9 and 13 Deans, respectively). Activities' effectiveness was usually unassessed, though some Deans used occasional surveys, data from health programs, student evaluations, and student feedback at meetings/events to help evaluate their programs.

### Focus groups

Our three hours of focus groups yielded little information about students' perceptions of the relationships between their personal and clinical health promotion practices; most students either had not considered this link, or had little to say about it. A few preclinical students reported that their personal wellness is generally linked to their competence as physicians, asserting that "if we sacrifice our own health from studying too long, staying up too late, stressing out too much about exams, we can't take care of other people if we don't watch our own health first." Several clinical students stated that wellness was difficult to achieve ("We're really stressed, basically"), and that having access to help/mentorship might help promote wellness for them: "I [would like] having a designated person to whom students can turn at any time. That would be a hotline . . . A counselor." Deans generally agreed with the concept of putting a mentoring support system in place. However, both students and deans see few resources in the medical schools directed toward student wellness and what programming that is offered is reactive and small in nature. Both the students and the deans discussed wellness in terms of stress and mental well-being, rather than including physical health factors such as nutrition and exercise. Students felt that the best way to teach prevention would be through skill development and role modeling from faculty who incorporate prevention into their practice. The deans proposed that prevention be integrated throughout the curriculum and not be offered as a separate course; students concurred that more prevention instruction would be optimal and acknowledged that a separate course gives the impression that the content is less important and optional.

### Site visits

We visited three medical schools with especially good and abundant practices around medical student health (Emory, Mercer, and Loma Linda Universities), and several other schools with some activities that seemed also to merit mention. These schools were selected for in-depth interviewing, with the best practices outlined in Table Five being used on medical school campuses.

## Conclusions

Prior literature [ref ] has typically examined limited populations of medical students regarding personal health promotion, with few assessments of student well-being or of the success of various interventions, so only limited conclusions can be drawn (a situation that will be improved with this and other publications from HD). However, some trends may be emerging, such as students' health practices being good in some spheres [2], but not being maintained in medical school [3] and residency [4,5], with an increase in alcohol consumption, and a decrease in socialization and exercise[6]. Poor medical student health habits also include maladaptive behaviors such as students going to school when sick, self-prescribing, and under-using medical care [7]. While medical students' positive health behaviors may be encouraged by their expanding knowledge and peer and role model support [2], some students may avoid treatment because of concerns that others' knowledge of their illness may place them in academic jeopardy [8].

Medical student and physician health is of inherent interest, but it is especially of concern because of the well-documented link between physicians' personal health practices and their patient counseling practices [1]. Despite the clear need in medical school for an emphasis on student wellness, the number of health promotion programs is declining[9,10]: competing demands for faculty time and financial resources are barriers to program implementation, and there is virtually no systematic study of the effects of such programs beyond our HD work with surveying students' counseling practices and validating these surveys with simulated patients (in review).

We found consistent support from both Deans and students for medical schools' encouraging healthy student behaviors, though modest follow-through on this support. Though students seemed to have thought little about the relationships between their own personal and clinical health promotion practices, we were especially impressed with the Deans' unanimity that faculty members should model healthy behaviors. The deans' support of the relationship between physicians' personal and clinical health practices, and concern about their institutions' acting on this relationship bodes well for the role of HD principles in the future of medical education. The correlation between students' and deans' responses suggests that deans understand well their students' health environments. If acted on, this finding (coupled with deans' beliefs that the environment can and should be improved) could create important positive changes in medical education and in disease prevention.

## Competing interest

The author(s) declare that they have no competing interests.

## Authors' contributions

EF co-developed the protocol, helped guide analyses, and drafted and revised the manuscript. JH co-developed the protocol, obtained funding, and helped edit the manuscript. LE co-developed the protocol, performed analyses, and helped edit the manuscript. All authors read and approved the final manuscript.

## Pre-publication history

The pre-publication history for this paper can be accessed here:


